# GH-mRNA expression and prediction of genetic improvement using selection index for body weight traits in turkeys

**DOI:** 10.5194/aab-69-411-2026

**Published:** 2026-08-17

**Authors:** Mohamed H. Khalil, Alaa A. Abd El-Motaleb, Manal M. Abdel-Rahman, Emad M. Amin, Ibrahim Abousoliman, Mostafa K. Shebl

**Affiliations:** 1 Department of Poultry Production, Faculty of Agriculture (El-Shatby), Alexandria University, Alexandria 21545, Egypt; 2 Desert Research Center, Department of Animal and Poultry Breeding, 1 Mathaf El-Matareya St., El-Matareya, Cairo 11753, Egypt; 3 Genetic Engineering and Biotechnology Laboratory, Plant Pathology Department (Genetic Branch), Faculty of Agriculture, Damanhour University, Damanhour 22511, Egypt; 4 Research Institute for Farm Animal Biology (FBN), Dummerstorf, Germany

## Abstract

This study aimed to evaluate the expected responses to a selection index based on body-weight-related traits to improve the productive performance of turkeys and to investigate the relationship between body weight and growth hormone (GH) gene expression. Two lines of local Baladi turkeys were used: a selected line (S), chosen for high body weight at 6 months of age; and an unselected random-bred control line (C). Both lines originated from the same randomly mated base population. A selection index incorporating body weight (BW), shank length (SL), and breast width (BRW) at 6 months of age was constructed. This index resulted in expected genetic gains per generation of 441.71 g for BW, 0.26 cm for SL, and 1.93 cm for BRW. Growth hormone gene expression in the selected line was significantly higher than in the control line for both sexes. In males, GH showed a relative gene expression of 1.43 in the selected line compared with 1.01 in the control line, while in females, the corresponding values were 1.76 and 1.31, respectively. Differences in GH-mRNA expression between the two lines indicate a positive association between gene expression level and body weight, suggesting that GH expression may be a useful molecular marker in genetic selection programs aimed at improving body weight in turkeys.

## 1 Introduction

Poultry is a vital source of food and income for farmers in many economic and social contexts. Poultry is a rich source of protein, essential amino acids, and micronutrients, which are crucial for human growth, muscle maintenance, and overall health (Sheffield et al., 2024). With output statistics reaching over 5.8 million tons of poultry meat in 2021 (FAO, 2023), or roughly 4.2 % of total poultry meat production, the global market for turkey meat that considered the main product of raising turkeys has experienced significant expansion (Kálmán and Szöllősi, 2023). The growing global demand for meat has prompted breeders and farmers to produce turkeys with rapid growth rates (Salter, 2017; Baldi and Gottardo, 2017). Therefore, the objectives of turkey breeding, which are to enhance production and lower costs, depend heavily on boosting growth and yield (Aslam et al., 2011; Begli et al., 2019; Abdalla et al., 2021). In turkey breeding, selection indices are widely used to combine multiple traits into a single score, enabling breeders to make informed decisions that balance competing objectives, such as improving growth while maintaining reproductive fitness. These indices are constructed using estimated breeding values derived from statistical models that account for heritability, genetic correlations, and economic weights assigned to each trait (Wellmann, 2023). The selection and application of selection indices for traits such as body weight, shank length, and breast width in turkeys are integral to modern poultry breeding programs, as these traits are directly associated with meat production, structural soundness, and overall economic value. Body weight is a primary selection criterion due to its strong correlation with meat yield. At the same time, shank length and breast width are important indicators of skeletal development and muscle mass, respectively. Selection indices are statistical tools that combine these traits into a single score, weighted by their heritability, genetic correlations, and economic importance, allowing breeders to optimize genetic progress across multiple traits simultaneously (Rajkumar et al., 2023). Local black Baladi turkey production continues to lag that of common varieties. To genetically characterize this flock and assess its genetic diversity for use in selection and breeding programs, further research is needed. The black Baladi turkey is the indigenous turkey breed of Egypt, predominantly reared under backyard and smallholder production systems throughout the Nile Delta and Upper Egypt. The breed is characterized by glossy black plumage, moderate body size, adaptability to harsh environmental conditions, and good resistance to local diseases. Although its growth rate and reproductive performance are generally lower than those of commercial turkey strains, it is valued for its meat quality and constitutes an important reservoir of indigenous genetic diversity (Amin, 2014; Mostafa, 2019). Traditionally, black Baladi turkeys have been maintained through uncontrolled natural mating, with selection based mainly on phenotypic traits such as body weight, fertility, hatchability, and survivability. Recent studies have demonstrated that selection for body weight and crossbreeding with commercial strains, such as white Nicholas, can improve productive performance while preserving the adaptive characteristics of the local breed. However, no nationwide structured breeding and conservation program has yet been established for this indigenous genetic resource (Amin, 2014; Abd El-Motaleb et al., 2025).

The importance of gene expression analysis has grown significantly, with the quantitative reverse transcription polymerase chain reaction (RT-PCR) being the method of choice for analyzing specific genes or performing mid-density gene expression analyses (Bustin et al., 2009; Wang et al., 2020). The somatotropic axis (GH-GHR-IGF) plays a central role in regulating growth and development in animals. The importance of GH and its ability to increase productivity in domestic mammals is well supported by research on some mammalian species. However, the way that GH functions in poultry development appears to be highly complex. The chicken growth hormone plays a crucial part in improving chicken performance, mostly by supporting growth processes. GH, a polypeptide hormone that affects several physiological processes including development, egg production, homeostatic regulation of feed intake, and reproductive activities, is synthesized and secreted by the pituitary gland (El-Tahawy et al., 2021; Zhu La et al., 2023; Mariandayani et al., 2023; Jakhrani et al., 2024). Moreover, GH's main effects include promoting the synthesis of proteins and nucleic acids, increasing the use of fats for energy production, controlling osmotic balance, and promoting reproduction (Lanes, 2016; Zhou et al., 2023; Yamamoto and Bando, 2023). Previously, growth-selected lines showed higher hepatic GH receptor binding than GH in both mature (Ellestad et al., 2019) and young (Antar et al., 2020) turkeys and hens (Vasilatos-Younken et al., 1990; Yang et al., 2022).

The objectives of this study were to analyze the expected responses to the selection index utilizing body weight traits for improving turkey growth and to investigate the association between gene (GH) expression and body weight of turkeys to be used as a genetic marker in selection programs.

## 2 Materials and methods

The present study was conducted at the Poultry Research Centre, Poultry Production Department, Faculty of Agriculture, Alexandria University, Egypt, during the breeding season of 2019. Molecular genetics was conducted at the Genetic Engineering and Biotechnology Laboratory, Plant Pathology Department, Genetic Branch, Faculty of Agriculture, Damanhour University, and the Desert Research Centre, Ministry of Agriculture.

### 2.1 Scheme for mating

A total of 193 birds were used in a random mating system to maintain the Egyptian local Baladi turkeys' base population (G0) (48 males and 145 females). The selected parents (13 males and 55 females) were picked from the base population based on their highest 6-month body weight to reproduce the selected line in the first generation (F1). The first generation (F1) of selection consists of the control (C) line, which was randomly selected from the base population, and the selected line (S), which was begun from the selected parents by mass selection for increased 6-month BW. At 7 months of age, eggs were pedigreed and collected daily for each pen. Four weekly hatches were used. At hatching, poults were pedigreed and wing-banded at 1 day old. Body weights (BWs) of birds at 0, 1, 2, 3, 4, 5, and 6 months of age were recorded individually, and body weight gain (g) during the period (0–6) months of age (BWG). At 4 months of age, birds were sexed. All experimental birds were reared under conventional floor-pen conditions with appropriate stocking density, ad libitum access to feed and clean drinking water, and standard health management practices, including routine vaccination and biosecurity measures. The classical standards for ration constituents were regarded for each age group. From hatching to 4 weeks of age, all chicks were fed a practical turkey pre-starter diet providing 28.7 % protein and 2807 kcal ME kg^−1^. After that, the birds were given rations according to a program comprising three feeding stages (starter diet providing 23 % protein and 3000 kcal ME kg^−1^, grower diet providing 21 % protein and 3100 kcal ME kg^−1^, and laying diet providing 18 % protein and 2800 kcal ME kg^−1^). The different rations used were formulated to meet all nutrient requirements as established by NRC (1994).

#### 2.1.1 Selection index

Selection index formulas ( I ) for body weight (BW), shank length (SL), and breast width (BRW) at 6 months of age in local Baladi turkeys were computed according to Cunningham (1969).

1 $$I=b_{1}\mathrm{BW}+b_{2}\mathrm{SL}+b_{3}\mathrm{BRW}$$

The relative economic values of studied traits were calculated according to Lamont (1991). This method estimated the economic values of traits using the heritabilities of the traits studied. Simply, it is an “equal progress” index, meant to make equal progress in selection by considering the heritability of each trait and using the inverse of the heritability as the weight.

The weighting factors ( b 's) of the original selection index were obtained by solving the following equation given by Cunningham (1969):

2 $$Pb=Gv,\;\text{to give}\;b=P^{-1}(Gv),$$

where P= phenotypic variances and covariances matrix, $P^{-1}=$ inverse of the phenotypic variances and covariances matrix, b= column vector of weighting factors, G= genetic variances and covariances matrix, and v= column vector of economic values.

Furthermore, according to Cunningham (1969), the other properties of the selection index were calculated as follows:

The standard deviation of the index ( $\sigma_{I}$ ) $=\sqrt{b^{\prime }Pb}$ where $b^{\prime }=$ transpose of the weighting factors column vector.

The standard deviation of the aggregate genotype ( $\sigma_{T}$ ) $=\sqrt{v^{\prime }Gv}$ where $v^{\prime }=$ transpose of the economic value column vector.

Correlation between the index and the aggregate genotype ( $R_{\mathrm{IT}}$ ) $=\frac{\sigma_{I}}{\sigma_{T}}$ .

The value of each trait in the index ( $V_{t}$ ) is the percentage reduction in the rate of total genetic gain if that trait is omitted from the index. It is equal:

3 $$V_{t}=100-\sqrt{\frac{b^{\prime }Pb-b_{i}^{2}/W_{ii}}{b^{\prime }Pb}}\times 100,$$

where $b_{i}^{2}=$ a square element of the column vector of weighting factors. $W_{ii}$ =a diagonal element of $P^{-1}$ .

The expected genetic gain ( $\Delta G_{i}$ ) in each trait after one round of selection on the index ( i=1 ) was obtained by solving the following equation:

4 $$\Delta G_{i}=(bG_{i}I)(i)(\sigma_{I}),$$

where $bG_{i}I=\frac{b^{\prime }G_{i}}{b^{\prime }Pb}=$ (regression of the trait on the index). i= selection differential in SD units. $\sigma_{I}=$ standard deviation of the index.

### 2.2 Statistical analysis

#### 2.2.1 Body weight at different ages:

Body weight data were analyzed using a linear mixed-effects model for repeated measurements. Line (selected and control), age (0, 1, 2, 3, 4, 5, and 6 months), and their interaction were included as fixed effects. In contrast, “bird” was included as a random effect to account for repeated measurements on the same individual. When significant effects were detected, pairwise comparisons among least-squares means were performed using the Duncan test (Duncan, 1955). In addition, linear regression analysis was conducted separately for each line to estimate the average monthly growth rate over the experimental period. Data were analyzed using the general linear model of SPSS software (IBM SPSS Statistics, 2021).

The following linear model was used:

5 $$Y_{ijkl}=\mu +L_{i}+A_{j}+(LA{)}_{ij}+B_{k}(L_{i})+\epsilon_{ijkl},$$

where $Y_{ijkl}=$ body weight, $L_{i}=$ line (selected or control), $A_{j}=$ age, L×A= interaction, $B_{k}(L_{i})=$ random effect of bird nested within line, $\epsilon_{ijkl}=$ residual error.

#### 2.2.2 Heritability estimates and genetic and phenotypic correlations

For the selected line (F1), heritability estimates, and genetic and phenotypic correlations among BW and body measurements: shank length (SL) and breast width (BRW) at 6 months of age were estimated based on paternal half-sibs using the following linear model:

6 $$Y_{\mathrm{ijkl}}=\mu +s_{i}+H_{j}+X_{k}+e_{\mathrm{ijkl}},$$

where $Y_{\mathrm{ijkl}}$ = observation for each dependent variable, μ= overall mean, $s_{i}$ = random effect of the i th sire, $H_{j}=$ fixed effect of the j th hatch, $X_{k}=$ fixed effect of the k th sex, and $e_{\mathrm{ijkl}}$ = random error.

The interaction between the fixed effects was excluded from the model because it was not significant. For early ages, the sex effect was excluded from the model.

The components of variance were estimated using PROC VARCOMP (procedure variance components) in SAS (2009) with the restricted maximum likelihood (REML) method. Heritability estimates were estimated using the following formulae (Becker, 1984):

7 $$h_{S}^{2}=\frac{4\sigma_{S}^{2}}{\sigma_{S}^{2}+\sigma_{W}^{2}},$$

where $\sigma_{S}^{2}=$ variance component of the sire, and $\sigma_{W}^{2}=$ variance component of error.

The standard error (SE) of the estimation in this study is given by Becker (1984) as follows:

8 $$\mathrm{SE}\left(h_{S}^{2}\right)=4\cdot \sqrt{\frac{2(1-t{)}^{2}[1+\left(k-1\right){]}^{2}}{k(k-1)(S-1)}},$$

where

$$t=\text{intraclass correlation}=\frac{\sigma_{S}^{2}}{\sigma_{S}^{2}+\sigma_{W}^{2}}.$$

K= average number of progenies per sire and calculated by the following formula:

$$k=\frac{1}{S-1}[N..-\frac{n_{i.}^{2}}{N..}].$$

S= number of sires, N..= total number of progenies, and $n_{i.}=$ number of progenies per sire.

Statistical analysis was also carried out according to the multivariate linear random model (MANOVA model) for all traits studied. Multivariate analysis of variance of the k traits was performed simultaneously to estimate the variance components of all effects for each trait and the pairwise covariances among them. These estimates were calculated using the MANOVA option of the GLM procedure in the SAS software package (Khattree and Naik, 2000; SAS, 2009; Holland, 2006). Phenotypic and genetic correlations were estimated using the following formulas (Becker, 1984).

The formula for estimating phenotypic correlation ( $r_{p}$ ) is

9 $$r_{p}=\frac{{\mathrm{cov}}_{p(xy)}}{\sqrt{\sigma_{p(x)}^{2}\cdot \sigma_{p(y)}^{2}}},$$

where $\sigma_{p(x)}^{2}=$ phenotypic component of variance for the trait x , $\sigma_{p(y)}^{2}=$ phenotypic component of variance for the trait y , and cov ${}_{p(xy)}=$ phenotypic component of covariance for the traits x and y .

The formula for estimating genetic correlation ( $r_{S}$ ) is

10 $$r_{S}=\frac{{\mathrm{cov}}_{S(xy)}}{\sqrt{\sigma_{S(x)}^{2}\cdot \sigma_{S(y)}^{2}}},$$

where $\sigma_{S(x)}^{2}=$ sire component of the variance for the trait x , $\sigma_{S(y)}^{2}=$ sire component of the variance for the trait y , and ${\mathrm{cov}}_{S(xy)}=$ sire component of covariance for the traits x and y .

An approximate standard error (SE) of genetic correlation is (Falconer and Mackay, 1996):

11 $$\mathrm{SE}\left(r_{A}\right)=\frac{1-r_{A}^{2}}{\sqrt{2}}\sqrt{\frac{\mathrm{SE}\left(h_{x}^{2}\right)\mathrm{SE}(h_{y}^{2})}{h_{x}^{2}h_{y}^{2}}}.$$

### 2.3 Molecular genetic analysis

#### 2.3.1 Muscle samples

At 6 months of age, 18 turkeys were selected for muscle sampling. The birds comprised two males and two females per replicate from the selection line (three replicates) and one male and one female per replicate from the control line (three replicates). All birds were maintained under identical housing, feeding, and management conditions throughout the experiment to minimize environmental variation. Birds were humanely slaughtered in accordance with the approved institutional animal care and use protocol. Immediately after slaughter (within approximately 5–10 min), about 1 g of the *Pectoralis major* (PM) muscle was aseptically excised, snap-frozen in liquid nitrogen, and stored at - 80 °C until RNA extraction and subsequent molecular analyses. The animals in this study were slaughtered at the certified slaughterhouse of the Faculty of Agriculture, Alexandria University. All experimental procedures, including the terminal slaughter for tissue collection, were reviewed and approved in accordance with the Alexandria University ethics code and national regulations (approval no. Alex. Agric. 092503205).

#### 2.3.2 RNA extraction and RT-PCR analysis, gene expression

Total RNA was extracted from samples using Trizol reagent (Qiagen Valencia, CA). The RNA samples were suspended in RNase-free water, and sample purity and concentration were measured on a UV/VIS spectrometer (pg T80, UK). For cDNA synthesis, 10 µ g of total RNA was reserved and transcribed with high-capacity cDNA reverse transcription kits according to the manufacturer's protocol (catalog number ABT009, Applied Biotechnology, Egypt). qPCR reactions were performed using the Rotor Gene (Qiagen Rotor Gene Q 5plex HRM, USA). A total of 1 µ L of cDNA served as a template in a 10 µ L PCR mixture containing 0.4 µ L each of forward and reverse primers from 10 µ m stocks and 2X Fast SYBR Green Master Mix (Trans Gen Biotech Co., LTD, China) and nuclease-free water to a final volume of 20 µ L.

The amplification of mRNAs was carried out by gene-specific primers designed using the primer-BLAST tool available on the National Centre for Biotechnology Information (NCBI) website (https://www.ncbi.nlm.nih.gov/tools/prime-blast/, last access: 5 August 2019). Primer sequences are presented in Table 1. The qPCR conditions were 95 °C for 3 min, followed by 45 cycles of 95 °C for 15 s, 60 °C for 30 s, and 72 °C for 30 s. In addition, at the end of each reaction, a melting temperature curve of every PCR reaction was determined. Data were normalized by β -actin expression as a reference gene in each sample (Table 1). The relative expression of the growth hormone gene was calculated using the $2^{-\Delta C_{t}}$ method, normalized strictly to the β -actin housekeeping gene ( $\Delta C_{t}=C_{t}$ , target- $C_{t}$ , reference) (Livak and Schmittgen, 2001).

**Table 1:** Primer sequence for qPCR.

Gene	Forward/reverse primer (5^′^-3^′^)	Product size	GenBank
		(bp)	accession no.
GH^∗^	F: 5^′^ ATCCACCTGCGCAACGA 3^′^ R: 5^′^ CCTTGTGCAGATCCTTCTTGAA 3^′^	85	NM_204359
β -actin	F: 5^′^ AGACATCAGGGTGTGATGGTTGGT 3^′^ R: 5^′^ TCCCAGTTGGTGACAATACCGTGT 3^′^	125	NM_205518.1

^∗^ GH = growth hormone.

The GH primer pair was designed to amplify a region located entirely within Exon 5 of the *Gallus gallus* growth hormone (GH) mRNA transcript (GenBank Accession No. NM_204359.2). The β -actin (ACTB) primer pair was designed to amplify a region located entirely within Exon 2 of the coding sequence of the *Gallus gallus* β -actin mRNA transcript (GenBank Accession No. NM_205518.2).

## 3 Results

### 3.1 Body weight at different ages

**Figure 1:**
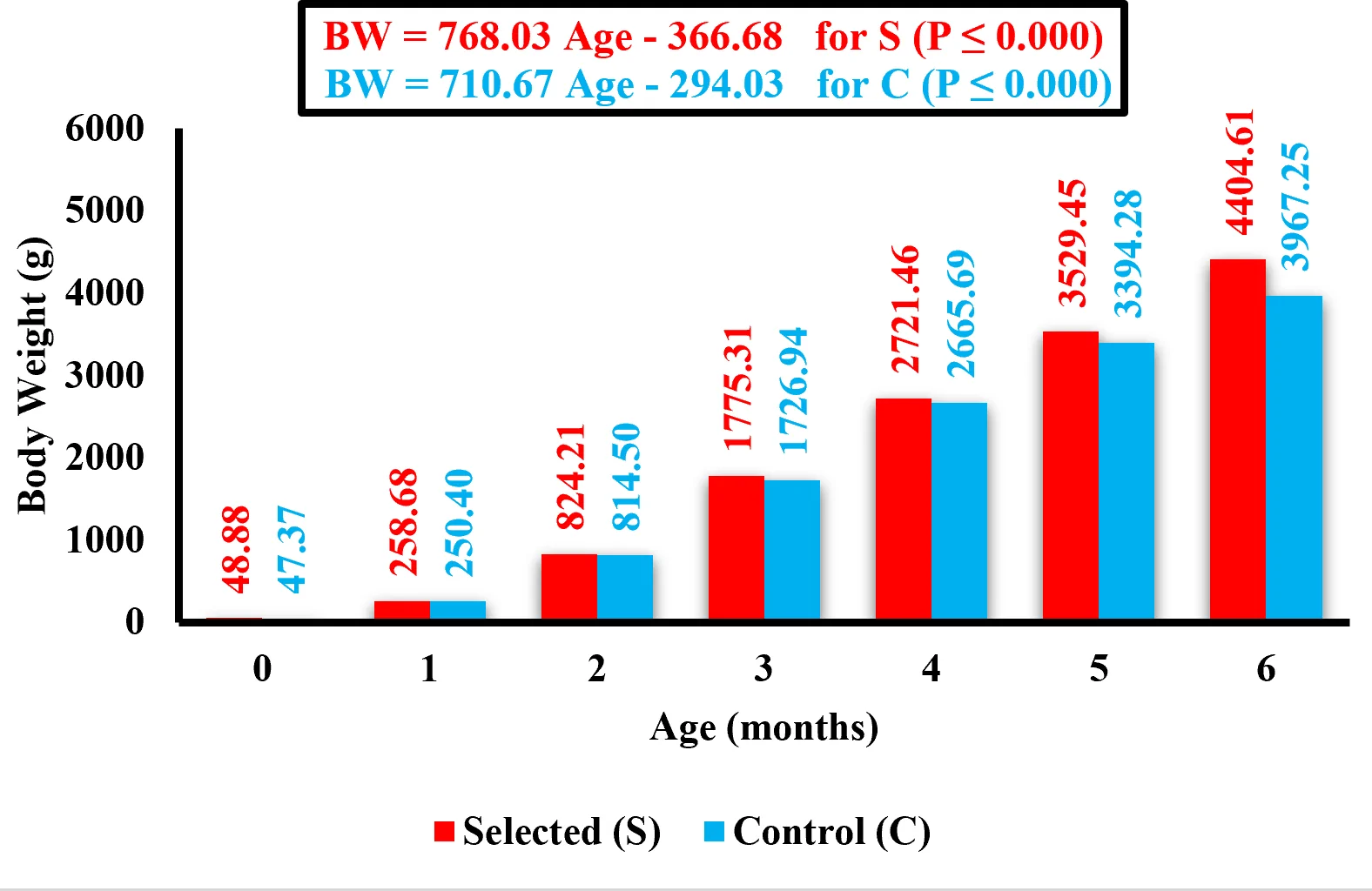
Means of BW (g) at different ages (months) for S and C lines along with regression analysis.

The mean body weight (BW) of Baladi turkeys at 0, 1, 2, 3, 4, 5, and 6 months of age in the selected and control lines is presented in Fig. 1. In the selected line, the mean BW values at the respective ages were 48.88, 258.68, 824.21, 1775.31, 2721.46, 3529.45, and 4404.61 g, whereas the corresponding values in the control line were 47.37, 250.40, 814.50, 1726.94, 2665.69, 3394.28, and 3967.25 g. Body weight increased significantly ( P≤0.001 ) with age in both the selected and control lines. Although the selected line consistently exhibited higher mean BW than the control line throughout the study, the difference between lines was statistically significant ( P≤0.01 ) only at 6 months of age, when birds from the selected line were significantly heavier than those from the control line (4404.61 vs. 3967.25 g). Regression analysis further demonstrated a significant linear increase ( P≤0.001 ) (see Table 2) in BW with age, with estimated growth rates of 768.03 g per month and 710.67 g per month for the selected and control lines, respectively, over the 0–6-month growth period. A highly significant difference in body weight was also observed between sexes. Males had substantially greater body weight than females at 6 months (5599.07 vs. 3419.71). These results demonstrate a pronounced sexual dimorphism in body weight, with males weighing approximately 63.7 % more than females at 6 months of age (Table 2).

### 3.2 Body measurements at 6 months of age

At 6 months of age, shank length (SL) and breast width (BRW) were significantly greater in the selected line compared with the control line (Table 3). Mean SL values were 15.93 and 15.11 cm for the selected and control lines, respectively, while corresponding BRW values were 29.17 and 28.48 cm. Phenotypic correlations between BW and SL and between BW and BRW were 0.58 and 0.56, respectively. Genetic correlations were 0.69 for BW–SL and 0.97 for BW–BRW (Table 4). Male turkeys showed significantly higher SL and BRW values than females (Table 3).

**Table 2:** Means and standard error (SE) of body weight (g) of turkeys at 6 months of age by line and sex.

Line & sex	N	Body weight	
		(6 months)	
		Mean	SE
Selected	131	4404.61^a^	67.80
Control	39	3967.25^b^	126.06
Sig		****	
Males	69	5599.07^*α*^	133.62
Females	101	3419.71^*β*^	63.39
Sig		****	

^****^ P≤0.0001 . Different letters (small and Latin) in the same column indicate significant differences ( P≤0.05 ).

**Table 3:** Means and standard error (SE) of shank length and breast width (cm) at 6 months of age of turkeys by line and sex.

Line & sex	N	Shank length		Breast width	
		(cm)		(cm)	
		Mean	SE	Mean	SE
Selected	131	15.93^a^	0.13	29.17^a^	0.24
Control	39	15.11^b^	0.28	28.48^b^	0.46
Sig		∗		∗∗ '	
Males	69	16.79^*α*^	0.15	31.20^*α*^	0.45
Females	101	15.03^*β*^	0.13	27.52^*β*^	0.36
Sig		**		**	

^∗^ P≤0.05 . ^**^ P≤0.01 . Different letters (small and Latin) in the same column indicate significant differences ( P≤0.05 ).

**Table 4:** Heritability estimates (diagonal), phenotypic correlations (above diagonal), genetic correlations (below diagonal), means, phenotypic standard deviation ( $\sigma_{p}$ ), genetic standard deviations ( $\sigma_{g}$ ), and economic values ( v ) were used to construct the selection index.

Traits	Traits			N	Means	$\sigma_{p}$	$\sigma_{g}$	V
	BW	SL	BRW					
Body weight, g (BW)	0.46 (0.25)	0.58^**^	0.56^**^	131	4404.61	776.01	526.32	1.00
Shank length, cm (SL)	0.69 (0.27)	0.21 (0.18)	0.38^**^	131	15.93	1.54	0.71	2.19
Breast width, cm (BRW)	0.97 (0.05)	0.37 (0.06)	0.64 (0.30)	131	29.17	2.76	1.88	0.72

^**^ P≤0.01 . Within the parentheses are the standard errors.

### 3.3 Selection index analysis

**Figure 2:**
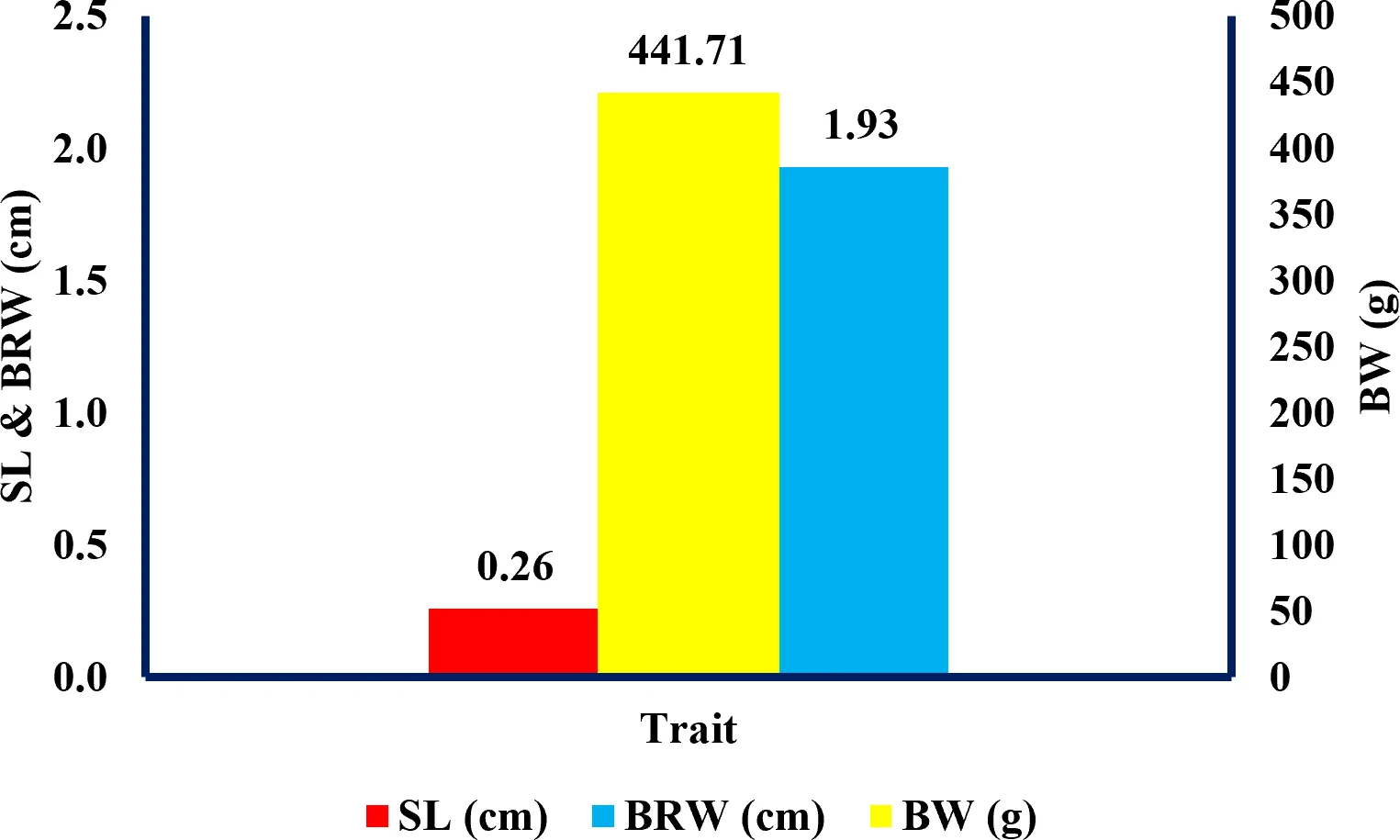
Expected genetic gain for the studied traits using the selection index.

The selection index included BW, SL, and BRW measured at 6 months of age. Estimates of heritability, correlations, means, phenotypic and genetic standard deviations, and relative economic values are presented in Table 4. Regression coefficients of traits on the index ( $b_{\mathrm{Gi}}I$ ) were 0.99561 for BW, 0.00058 for SL, and 0.00435 for BRW (Table 5). The corresponding weighting factors (b) were 0.30, - 56.64, and 113.00 for BW, SL, and BRW, respectively. The omission of BRW from the index resulted in the highest reduction in overall genetic gain (18.57 %), followed by BW (7.69 %), whereas SL showed the lowest contribution (1.28 %). The standard deviations of the index ( $\sigma_{I}$ ) and aggregate genotype ( $\sigma_{t}$ ) were 443.65 and 528.92, respectively, and the correlation between the index and aggregate genotype ( $r_{\mathrm{IT}}$ ) was 0.84 (Table 6). The expected genetic gains per generation were 441.71 g for BW, 0.26 cm for SL, and 1.93 cm for BRW (Table 6 and Fig. 2).

**Table 5:** Regression of each trait on the index ( $b_{\mathrm{Gi}}I$ ), weighting factors ( b 's), and value of each trait ( $V_{t}$ ) in the index.

Traits	$b_{\mathrm{Gi}}I$	b 's	$V_{t}^{*}$ (%)
Body weight, g (BW)	0.99561	0.30	7.69
Shank length, cm (SL)	0.00058	- 56.64	1.28
Breast width, cm (BRW)	0.00435	113.00	18.57

^∗^ Percent reduction in the rate of overall genetic gain if that trait is omitted.

**Table 6:** The standard deviation of the selection index ( $\sigma_{I}$ ), the aggregate genotype ( $\sigma_{t}$ ), the correlation between the index and aggregate genotype (true breeding value) ( $r_{\mathrm{IT}}$ ), and the expected genetic gain ( ΔG ) for the studied traits using the selection index.

Traits	$\sigma_{I}$	$\sigma_{t}$	$r_{\mathrm{IT}}$	ΔG
Body weight, g (BW)	443.65	528.92	0.84	441.71
Shank length, cm (SL)				0.26
Breast width, cm (BRW)				1.93

### 3.4 Growth hormone (GH) gene expression

**Figure 3:**
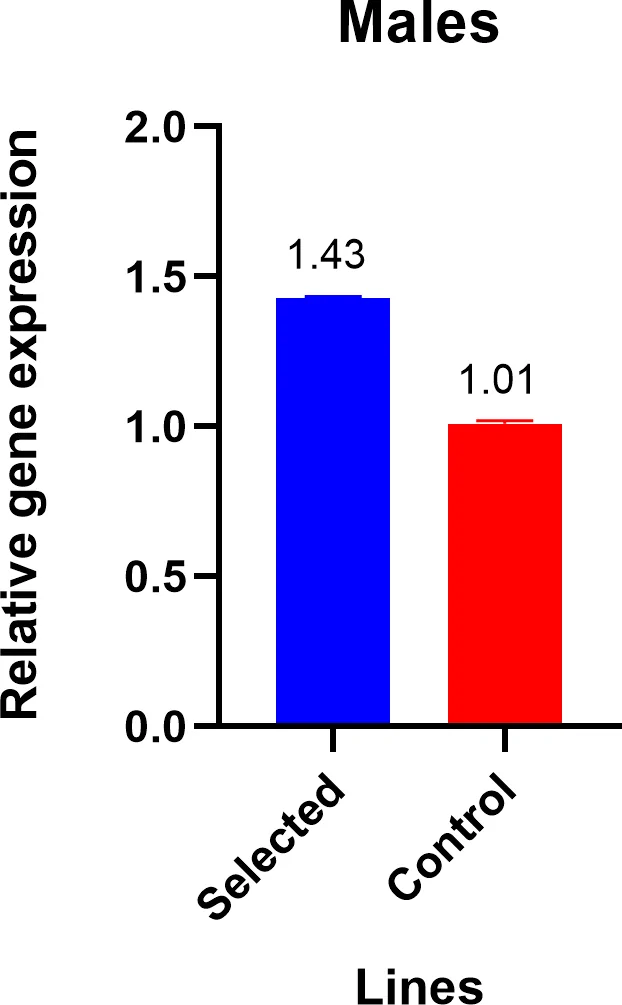
mRNA expression analysis of the GH gene using qRT-PCR for the selected and control lines of turkey males.

**Figure 4:**
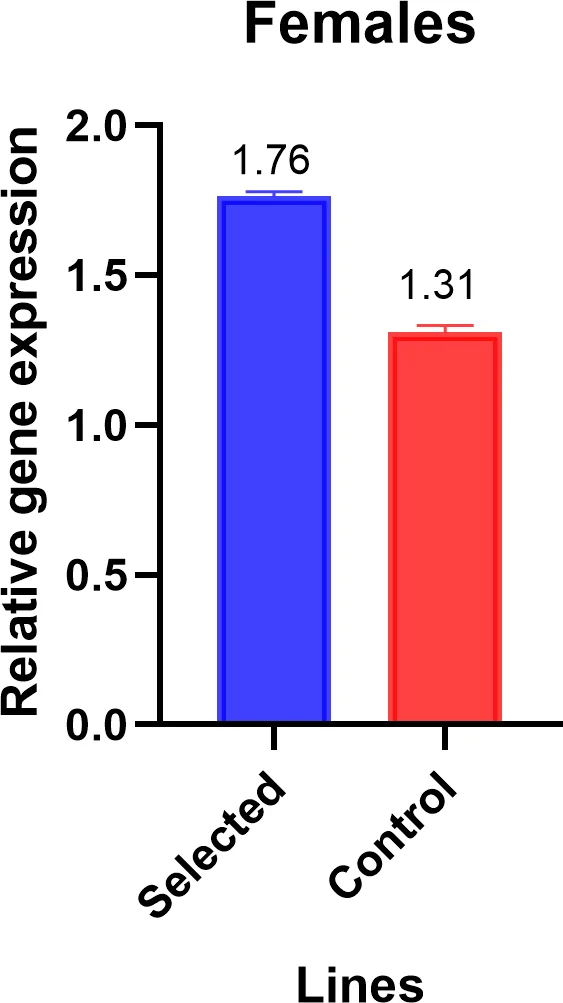
mRNA expression analysis of the GH gene using qRT-PCR for the selected and control lines of turkey females.

GH-mRNA expression was evaluated in the breast muscle of selected and control lines in both sexes. GH expression was significantly higher in the selected line than in the control line in both males (1.43 vs. 1.01 relative gene expression; Fig. 3) and females (1.76 vs. 1.31 relative gene expression; Fig. 4). Females also exhibited significantly higher GH expression than males. The selected line, which had higher body weight at 6 months of age, also showed higher GH expression than the control line.

## 4 Discussion

The consistently higher body weight observed in the selected line across all ages confirms the effectiveness of individual selection for BW at 6 months of age. Similar trends were reported by Sabra et al. (2017), Ogah (2011), and other studies, indicating that sustained genetic improvement in BW can be achieved through targeted selection.

The significant positive phenotypic and genetic correlations between BW and body measurements (SL and BRW) suggest that these traits share common genetic control. This explains the correlated response observed in body dimensions following selection for BW. The strong genetic correlation between BW and BRW (0.97) indicates that BRW is a particularly reliable indirect selection criterion for improving body weight and meat yield.

Selection index analysis demonstrated that BRW contributed the most to overall genetic gain, followed by BW, while SL contributed the least. This highlights the importance of incorporating BRW into breeding objectives to improve growth and carcass traits. Similar findings were reported by Oblakova (2006), Aslam et al. (2011), and Rajkumar et al. (2023), who emphasized the superiority of selection indices over single-trait selection.

The high accuracy of the selection index ( $r_{\mathrm{IT}}=$ 0.84) confirms its effectiveness in ranking individuals based on aggregate breeding value. The expected genetic gains indicate that selection using this index can simultaneously improve BW and associated body measurements, provided sufficient genetic variation is present in the base population.

The higher GH-mRNA expression observed in the selected line supports the hypothesis that molecular genetic mechanisms underpin phenotypic improvement in growth traits. The growth hormone plays a central role in regulating growth rate, muscle development, and metabolism through the GH/IGF-1 axis. The observed sex differences in GH expression, with higher levels in females, are consistent with previous reports (Numair et al., 2023; Sinpru et al., 2021).

Previous studies have reported associations between GH gene polymorphisms, GH-mRNA expression, and growth-related traits in poultry (Ghelghachi et al., 2013; Jia et al., 2018; El-Attrouny et al., 2020; Sinpru et al., 2021; Mohamed Ali et al., 2024). However, gene polymorphisms and gene expression represent different biological aspects and should be interpreted separately. The higher GH-mRNA expression observed in the selected line is consistent with its superior growth performance, although the present study does not establish a causal relationship between selection and GH gene upregulation. The higher GH expression may reflect differences associated with the selected phenotype rather than a direct effect of selection itself. Given the well-established role of the GH/IGF-1 axis in regulating muscle development and growth, the observed expression pattern supports the hypothesis that GH may contribute to growth differences between the two lines.

Overall, the findings demonstrate that traditional selection improved phenotypic performance in the selected turkey line and was associated with higher GH-mRNA expression. These results suggest that phenotypic improvement and differences in GH-mRNA expression occurred concurrently; however, the present study does not establish a causal relationship between selection and changes in GH expression. Further studies are needed to clarify the biological mechanisms underlying this association and to evaluate the potential of GH expression as a molecular indicator in turkey breeding programs.

## 5 Conclusion

This study demonstrates that traditional breeding using a selection index effectively improved growth performance and economically important traits in the selected turkey line. The observed differences in GH-mRNA expression between the selected and control lines suggest that GH-mRNA expression is associated with growth performance, although its value as a molecular marker requires further validation. Integrating conventional phenotypic selection with molecular analyses may provide additional insights into the biological mechanisms underlying genetic improvement.

## Data Availability

The data sets used in this article can be requested from the corresponding author.
